# Identifying Barriers and Facilitators to Care for Infants with Bronchopulmonary Dysplasia After NICU Discharge: A Prospective Study of Parents and Clinical Stakeholders

**DOI:** 10.21203/rs.3.rs-3377817/v1

**Published:** 2023-10-09

**Authors:** Joanne Lagatta, Melissa Harris, Rachel Cusatis, Margaret Malnory, Sara Dawson, Girija Konduri

**Affiliations:** Medical College of Wisconsin; Medical College of Wisconsin; Medical College of Wisconsin; Med College of Wisconsin & Children’s Research Institute of Children’s Hospital of Wisconsin; Med College of Wisconsin & Children’s Research Institute of Children’s Hospital of Wisconsin; Med College of Wisconsin & Children’s Research Institute of Children’s Hospital of Wisconsin

**Keywords:** prematurity, neonatal intensive care, social determinants of health, bronchopulmonary dysplasia

## Abstract

**Objective:**

Understand barriers and facilitators to follow-up care for infants with bronchopulmonary dysplasia (BPD).

**Methods:**

Qualitative study of parents and clinical stakeholders caring for infants with BPD. The interview guide was developed by a mother of a former 23-week preterm infant, neonatologist, pulmonologist, nurse, and qualitative researcher. Purposive sampling obtained a heterogenous sociodemographic and professional cohort. Subjects discussed their experience with BPD, barriers to care, caregiver quality of life and health education. Interviews were audio-recorded, transcribed and coded. Thematic analysis was used.

**Results:**

Eighteen parents and 20 stakeholders completed interviews. Family-level themes included pragmatic barriers like transportation being multi-faceted; and caregiving demands straining mental health. System-level themes included caregiver education needing to balance process needs with future trajectories; and integration of primary care, specialty care, and community supports.

**Conclusions:**

Individual and system barriers impact follow-up for infants with BPD. This conceptual framework can be used to measure and improve care.

## Background

Infants born extremely preterm are at high risk of developing bronchopulmonary dysplasia (BPD).(1–7) In the United States, 10,000 to 15,000 infants develop BPD each year.(8) BPD has major implications for young children and families as they transition home from neonatal intensive care; 70% of infants with BPD experience post-prematurity respiratory disease by 1 year of age.(9–11) Compared to other preterm infants, infants with BPD experience longer NICU stays and more re-hospitalizations.(12–14) Respiratory symptoms such as wheezing can persist throughout childhood leading to more medication use, outpatient and emergency care.(15–20) Durable medical equipment leads to frequent interactions with specialist healthcare providers, home nursing and equipment companies. Although it has been shown that these complex health needs negatively impact parent health-related quality of life, it is not known how healthcare supports may serve as barriers or facilitators for families caring for infants with BPD as they transition home from the NICU.(15–20) A deeper understanding of child and family experiences with post-discharge healthcare utilization for infants with BPD would inform better discharge practices for infants with this complex condition.

Multidisciplinary supports after NICU discharge are intended to improve outcomes for infants; however, specialty care can only improve outcomes if families can access supports. Unfortunately, families of infants with BPD may face significant barriers to care. Prematurity disproportionately impacts families experiencing socioeconomic disadvantage, who in turn experience barriers to accessing the outpatient follow-up care that could improve health outcomes.(21–24) Socioeconomic and racial disparities in NICU care have been increasingly highlighted, and likely contribute to disparities in parent discharge preparation for high-needs infants.(25–29) Health systems vary widely in clinic staffing and care coordination.(21, 30–33) Research to understand the nuances to these potential barriers to care for infants with BPD would help inform a conceptual framework for measurement, research and improvement.

The goal of this study was to better understand barriers and facilitators to care among stakeholders and parents of infants of BPD, in order to understand how to improve discharge preparation and follow-up care for infants with BPD.

## Methods

### Recruitment and Study Population

This was a qualitative study using semi-structured interviews of parents and clinical stakeholders caring for infants with BPD, following consolidated criteria for reporting qualitative research.(34) Parents and clinical stakeholders were identified for potential participation by members of the research team with clinical or professional knowledge of potential participants (MH, MM, SD, JL). Purposive sampling was used to obtain a heterogenous sample across sociodemographic characteristics for parents and across professional roles for stakeholders. Parents were recruited in person from the NICU, BPD pediatric pulmonology clinic and developmental follow-up clinic, with clinical team members introducing the interviewer to families. Parent-infant dyads who met the following inclusion criteria were invited to participate in the study: 1) infant born < 32 weeks; 2) diagnosed with BPD defined by receipt of respiratory support at 36 weeks’ corrected age in the NICU; 3) at least one English speaking parent; 4) no tracheostomy (due to the different level of complexity and team involvement for that patient population). Stakeholders were invited by email. Eligibility included being either a healthcare provider caring for infants with BPD in the inpatient or outpatient clinical setting, or a staff member experienced with preterm infants and their families transitioning from hospital to home, such as a social worker, case manager, or community service professional (e.g., Special Supplemental Nutrition Program for Women, Infants, and Children professionals). Subjects were recruited until thematic saturation. The study was reviewed and approved as exempt by the institutional review board of the Medical College of Wisconsin. All participants provided verbal consent to participate. Interviews were offered in person, via phone or computer; 2 interviews were completed in person and the rest were completed over the phone with audio recording. Parents signed a HIPAA waiver for viewing the infant health record and received a $25 gift card for participation.

### Interview Content

Interviews were conducted individually by a research assistant trained in qualitative interview methods. The interviewer is a doctoral student and mother of a former 23-week preterm infant, working in collaboration with a neonatologist, pediatric pulmonologist, nurse, and qualitative research expert to construct the interview guide. Stakeholders and parents were told about the members of the interview team and the background of the interviewer. Interview guides followed a similar format for parents and stakeholders (Table 2). For parents, content areas included 1) parent’s personal history leading up to their child’s preterm birth; 2) the child’s health symptoms over time; 3) perspectives on health services including gaps in care, barriers, facilitators, and resources; 4) quality of life including social support, physical and mental health. For stakeholders, we asked about 1) professional roles related to caring for preterm infants with BPD; 2) perspectives on important symptoms and issues for patients with BPD (for clinical stakeholders); 3) perspectives on key supports for infants with BPD, including challenges to accessing care and providing condition-specific health education; 4) issues regarding family quality of life and social support. The interview guide was tested with a sample of parents and stakeholders before beginning the study.

### Data Analysis

Interviews were audio-recorded, transcribed verbatim and coded using MAXQDA 2022 qualitative analysis software; field notes were made after each interview. Transcripts were not returned to interviewees for review, but a preliminary summary of findings was made available to interested participants for feedback. Four members of our team, representing both clinical and methodological expertise, read two transcripts and discussed a high-level summary of themes to develop a preliminary codebook. To facilitate team-based coding, our codebook included a code name, a definition, inclusion and exclusion criteria to help distinguish codes from one another, and examples. The final codebook included stakeholder role (including direct and indirect care), pragmatic barriers (including transportation, language, mistreatment or discrimination), resources, education (including content and delivery), system (coordination, communication and provider access), policy (including reschedule or late-arrival policies), family capacity (including social support, competing caregiving demands, isolation, finances and childcare), and addressing barriers or gaps in care (including screening and timing of barriers). We used a three-pronged analytic coding strategy which included open, axial, and selective coding.(35, 36) Interviews were split equally for primary coding by two team members (MH and MM); half of transcripts were coded twice by both coding team members (MH and MM) to check for consistency. A third team member (JL) reviewed all transcripts and codes to identify and discuss any potential discrepancies. Coded interviews were analyzed using thematic analysis to identify emerging themes.(37) A grounded theory approach was used to examine concepts and themes within the data and develop an explanatory framework of barriers and facilitators to care as identified by stakeholders and parents of infants with BPD.(36, 38)

## Results

A total of 25 parents were approached for participation; 25 agreed to participate, and 18 completed interviews with the remaining 7 not completed due to scheduling difficulty and thematic saturation. A total of 22 stakeholders were approached for participation; 20 completed interviews. Parents represented a range of education, income, race and ethnicity. Stakeholders represented a range of specialty and primary care, social work, care coordination, and community services (Table 1). Each interview occurred only once. Interviews ranged from 25–60 minutes to complete.

**Figure 1:**
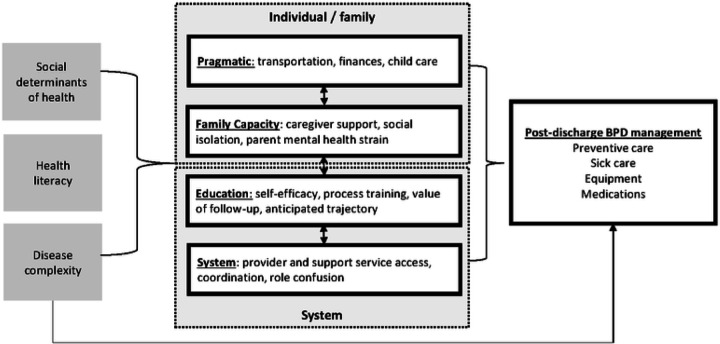
Conceptual framework of barriers to care for infants with bronchopulmonary dysplasia after discharge. Themes and examples were identified by thematic analysis of interviews with both parents and clinical stakeholders.

Interviews of parents and stakeholders identified four primary themes (Fig. 1). Family-level themes included multifaceted social needs and family functioning affecting navigation of NICU and post-discharge care. System-level themes included caregiver education needing to balance tangible process needs and broader expectations, and communication and coordination of services.

### The multifaceted nature of social needs exacerbates barriers to screening and service referrals

Pragmatic barriers to attendance in the NICU and post-discharge appointments were commonly described by both parents and stakeholders (Table 3). Transportation was a major barrier to attending follow-up appointments and was described by both parents and stakeholders as multi-faceted. Transportation barriers included individual or family barriers such as not having a car, not having money to afford gas, and having to arrange childcare for siblings in order to attend clinics; they also included system barriers such as unreliable transportation services and appointment inflexibility in the case of late arrivals. Stakeholders felt that responding to even a specific barrier like transportation was often nuanced. Inpatient-based stakeholders noted that resource referrals benefitted from in-person communication to properly assess and refer to resources, whereas outpatient teams generally described involvement after a pattern of inability to make appointments. Primary care teams felt that transportation may be more of a barrier to specialty services further from home than to their clinics.

Most parents felt that the NICU and clinics do well in addressing needs such as transportation. While receiving resources was helpful in reducing barriers, some parents described wanting to be aware of resources sooner. Stakeholders agreed that there was a lack of awareness of available resources, which may be due in part to limitations in systematic screening for social needs. Reasons provided for limited systematic screening included changing needs over time which may not be uncovered during initial screenings, a perceived overwhelming degree of needs, and insufficient supportive resources.

### Family functioning: Competing health and caregiving demands strain family and mental health

Parents commonly expressed challenges balancing the competing life demands of employment and family in addition to the caregiving needs of an infant with a complex health condition (Table 3). Specific barriers included not having childcare to attend appointments, the regimented daily requirements for infants with medical equipment along with needs of other siblings, and the desire to reduce infectious exposures limiting the availability of help. When parents needed to alternate time in the NICU with other family or job demands, this exacerbated family functioning stress after discharge because other family members’ lack of training in the NICU meant there were fewer prepared caregivers to help with an infant’s needs at home. Some parents described not working after NICU discharge to be able to solely care for their infant, or having opposite shifts of their significant other or family, as a solution to sharing care needs. Parents also discussed the family and mental health strain caused by social isolation and competing demands for such a long time. Some parents expressed trauma related to their infant’s care needs, including guilt of preterm delivery, being back in the hospital, seeing that the baby “isn’t normal,” and being cognitively but not emotionally prepared for the workload required. Facilitators to family functioning included flexibility to limit work, ability and expectations to share sibling care, and prior experiences dealing with healthcare. More modifiable facilitators included trust in nurses or healthcare providers to gain support, networking opportunities with other families, connection to resources to reduce financial barriers, and ability to seek mental health support.

Stakeholders similarly identified that families were challenged with competing life demands such as balancing work and childcare responsibilities, and the strain placed on individuals and family relationships. Some stakeholders expressed that fear of the healthcare system after their experiences with pregnancy and the NICU can lead to a need to get away from the hospital and potentially avoid subsequent healthcare system exposure, but can also lead to fear of leaving the hospital and hypervigilance about certain symptoms. Clinical stakeholders expressed the importance of family functioning in supporting child health, but most acknowledged that they do not universally ask about these issues due to available time and resources. From a stakeholder perspective, facilitators to improving family function included better resources to support both pragmatic needs and psychosocial support.

### Caregiver education needs to balance tangible process needs and broader expectations

Parents overall described receiving excellent instruction focused on equipment management and infant care (Table 4). Facilitators to caregiver self-efficacy included home nurses, teaching materials, being present in the NICU for more preparation time, and reassurance from supportive providers. Themes in educational gaps included anxiety about the learning curve; many parents felt that their how-to instruction was good, but that the mental preparation was nerve-wracking. Many parents noted the difference between observing skills demonstrated in the NICU and taking primary responsibility at home. A frequent request was to offer more second-caregiver education; many mothers reported being the only person trained to use equipment, do feeding, and give medications, which then caused difficulty after discharge because of competing needs and delay in other caregivers being able to provide support. Another theme was a request for more education on parenting and life after the NICU, including what to watch for in childhood, and managing anxiety.

Stakeholders identified several discrete educational needs. These included process needs such as understanding equipment, handling medication refills, and knowing who to call after discharge; acute care education such as mitigating viral exposures and actions prior to seeking emergency care; and preventive care needs such as the role of specialty follow-up. Specific to infants with BPD who have had ongoing intermittent desaturations in the NICU for weeks to months, a gap was also noted in understanding the importance of appropriate oxygen saturations. Expected trajectory was identified as a gap; examples included expectations for school, exercise limitation, duration of respiratory support, and when childcare outside the home could be safer. Stakeholders emphasized that health literacy and stress were major barriers to educating families around infant diagnosis and care needs.

### Care coordination and communication: important and constrained by system limitations

Parents thought that scheduling post-discharge appointments was not a challenge in itself; however, clustering appointments together was difficult given limited appointment availability (Table 4). Parents also identified communication between teams as a system barrier to obtaining care; examples included communication between specialists and pediatrician, or between multiple teams and the family, as to clear delineation of roles and the “why” for each visit. Some parents felt that their primary care provider was reluctant to interfere with specialist recommendations so as not to provide conflicting information, and as a result the families were not sure of their pediatrician’s comfort with their child’s medical needs. Parents identified the need to advocate for their child across system gaps such as lack of follow-through with scheduling between teams or resources; they identified their own background or education as a facilitator for those with a background in healthcare, and they identified healthcare providers’ insensitivity at times to family challenges as a barrier to being able to advocate strongly. Those who participated in care coordination programs found it helpful for scheduling and team communication.

Stakeholders also highlighted that specialty provider availability can pose barriers to care. The use of care coordination services was seen as a potential facilitator to help alleviate the challenges of multiple appointments, but limitations included that families may not appreciate that benefit immediately. Another issue that stakeholders highlighted was conflicting recommendations between providers leading to confusion; examples included recommendations on nutrition issues or who to call with acute respiratory symptoms.

Some constructs were present across multiple individual and system themes (Fig. 1). Social determinants of health crossed all levels of barriers to care, in that financial barriers limited access to in-person communication to enable education of primary and secondary caregivers, which perpetuated problems with extended family support and abilities to advocate for better care coordination. Health literacy was commonly identified by stakeholders as an educational barrier, whereas parents were more likely to describe it indirectly as a barrier or facilitator in advocating for coordination of care. Complexity of needs including medications, equipment or frequent appointments impacted need for education and care coordination, but also was mentioned as a contributor to family and mental health strain.

## Discussion

The goal of this study was to identify barriers and facilitators to care among stakeholders and parents of infants of BPD, in order to understand how to improve discharge preparation and follow-up care specific to this condition. Our main findings were: 1) the multi-faceted nature of social needs exacerbates barriers to screening and resource referrals; 2) competing health and caregiving demands strain family functioning and mental health; 3) caregiver education needs to balance tangible process needs and expected trajectories within the context of stress and variable health literacy; 4) care coordination opportunities are both important and constrained by system limitations in pediatric subspecialty care.

Given the lengthy NICU stays, and frequent outpatient appointments required of infants with BPD, it is not surprising that pragmatic barriers such as transportation were a major theme impacting access to care. Both parents and clinical stakeholders recognized that supporting needs such as transportation were multi-faceted, and both parents and clinical stakeholders recognized the limitations of community resources in supporting these needs. In response to these barriers and limitations, parents more frequently expressed desires to be provided with earlier, ongoing screening to increase their awareness of resources. Stakeholders were more likely to prefer in-person introductions to establish rapport and understand a family’s needs in more detail; after that introduction, subsequent resource referrals were more likely to occur after a pattern of nonattendance or an explicit request. From a system standpoint, depending on in-person interaction to identify a need for assistance with getting to in-person appointments can delay or prevent understanding of families’ needs and getting them connected to resources. Systematic screening for social determinants of health is increasingly recognized as an important way to improve health outcomes.(39–43) The application of systematic screening for resource needs has been more widely demonstrated in outpatient settings; adaptations to the inpatient setting are emerging more recently.(40, 41) To support infants with BPD preparing for complex discharge home from the NICU, it may be helpful to consider both inpatient and outpatient scenarios to help families envision their upcoming needs and arm themselves with appropriate resources.

Both parents and stakeholders recognized the family and mental health strain resulting from competing caregiving demands. While some aspects of social support are not modifiable by a health care team, it was encouraging to observe that parents identified several actionable ways to increase family capacity. Identifying ways to increase educational offerings such that additional caregivers do not need to be trained solely by the parent who was able to be at the bedside could be an important facilitator of post-discharge family capacity, since the differential in caregiver comfort causes frequently reported family strain. Videos or multi-media offerings could assist family caregivers who are not able to be present for all hospital and clinic-based education. Anticipatory support for mental health including the trauma of preterm birth and emotions surrounding a baby who requires significant post-discharge medical supports are important topics to address before and after NICU discharge. Most NICUs have limited social work and psychology resources to assist families, but this limitation is likely even more pronounced in the outpatient setting. Systematic outpatient psychosocial support as part of NICU follow-up is critical especially for fragile infants such as those with BPD, since parents commonly describe that nothing could prepare them for the emotional toll of home caregiving despite their cognitive preparation.

While both parents and stakeholders generally identified good education to help teach processes related to their infants’ care needs, they all also agreed on the gap in education on expected needs of a child with BPD over time. Even for infants with multiple outpatient clinical and community support resources, the weeks and months spent in the NICU before going home may be the majority of time available for direct hands-on family caregiver education. This time is spent learning infant care, which can be overwhelming even before adding the complex needs of an infant with BPD. Strategies to enhance anticipatory guidance about life after the NICU might include earlier identification of equipment and medication needs, and more proactive meetings with relevant outpatient specialists to understand early childhood needs. It should be noted that some themes emerged in duality, such that it was difficult to disentangle family functioning from educational themes that pose barriers to meeting the needs of families with a child with BPD. Ongoing psychosocial support and multi-media educational offerings are crucial to working effectively within the context of stress, rotating work schedules, and variable health literacy that limit the ability to process information.

Even in the context of perceived high quality specialty care, families and stakeholders noted system limitations to care coordination. Dedicated care coordination teams are helpful facilitators to complex care, but do not always enroll infants with BPD going home from the NICU. NICU-specific transition-home programs have been shown to be effective in reducing health care expenditures and readmissions but have not become widespread across NICUs.(44–47) Our findings were consistent with this literature. Parents who did not receive care coordination agreed that care coordination would improve barriers, and those who did receive care coordination felt it made attending appointments easier. However, even in the presence of a care coordination team, outpatient pediatric subspecialty availability can limit the ability to streamline appointments; multidisciplinary clinics may improve this system gap. Parents also perceived primary care physicians to have variably active involvement with their child’s specialty needs. Consensus recommendations for outpatient management of BPD do not currently highlight roles for the primary care provider versus specialist care or a recommended schedule for pulmonology follow-up, leading to a lack of clarity on best practices to guide care coordination.

Strengths of this study include an interview guide designed to elicit multiple levels of potential barriers to care, parallel interviews between both parents and clinical stakeholders, intentional sampling strategy to recruit a diverse range of participants, and analysis integrating the perspectives of a parent of a preterm infant, nurse, and physician. Our findings provide detail that can be used to understand opportunities for system interventions to improve care for infants with BPD. We acknowledge several limitations. The study was conducted at one health system, although we did explicitly recruit parents of infants cared for at multiple NICUs. Although our qualitative strategy allows for a detailed understanding of perspectives, and we interviewed until reaching thematic saturation, the study cohort may not be representative of the whole population. Although the interviewer self-identified as a parent of a preterm infant and not a direct healthcare provider, there may be some desirability bias affecting parent willingness to acknowledge barriers within the healthcare system. Although the interview guide was constructed by multidisciplinary members of the research team, the interviewer’s life experience as a parent of preterm infant with BPD may have led to researcher bias. Additionally, parents who are unaware of community resources or healthcare system differences may not be able to fully identify potential barriers to obtaining care for their infant. Clinical stakeholders employed by our healthcare system, although anonymous, may also experience desirability bias in discussing potential system limitations to care. They also may differ in their sense of frequency of barriers to care compared to families, as has been demonstrated previously. (48)

## Conclusion

In this qualitative study of parents and stakeholders, we identified discrete yet inter-related domains of barriers and facilitators to follow-up care for infants with BPD after the NICU. These include pragmatic social barriers, caregiver strain impacting family functioning, family function impacting educational needs, and opportunities and limits to care coordination for pediatric specialty care. Despite different perspectives, families and stakeholders converged on several themes that could improve care for infants with BPD in the transition home from the NICU. This framework can help inform measurement and quality initiatives to improve the process of discharge for infants with BPD.

## Figures and Tables

**Table 1.** Characteristics of parent and stakeholder participants.

Parent respondents (n=18)			Stakeholder respondents (n=20)	
Education	Have not completed high school	3	MD or APN, specialty care (pulmonary, complex care, NICU follow-up)	5
	High school graduate	3	MD or APN, primary care	5
	Some college	3	Social work (inpatient, outpatient)	3
	College or technical school graduate	9	Case management, nurse coordinator	3
	
Household income	<$20,000	5	Psychologist	1
	$20,000–40,000	3	Community services (WIC, birth to three, DME)	3
	
	$40,000–60,000	2		
	>$60,000	8		
	
Race and ethnicity	White	11		
	Black or African American	6		
	Hispanic, Latino or Spanish origin	1		
	Asian	1		
	American Indian or Alaska Native	1		
	
Sex	Woman	15		
	Man	3		
	
Disability status	None identified	14		
	Learning disability	4		
	Mental health concern	1		

**Table 2:** Sample interview prompts for stakeholders and parents.

	Stakeholder question prompts	Parent question prompts
Role	Can you tell me about yourself and your position? How do you interact with infants with BPD and their families? Can you think about infants or children who might benefit from your services but who aren’t getting it?	Can you tell me about yourself? (living situation, caregiving responsibilities before baby’s birth) What experience did you have dealing with the healthcare system before [name]’s birth?
Respiratory experience?	What are the most common issues symptoms that patients with BPD What do you think are the most important to your patients?	What important symptoms do/did you observe when your child was bothered by breathing problems? How has that changed over time? Can you tell me about your experience with healthcare providers asking about your child’s breathing?
Perspective on services	What do you see as the biggest challenge to assessing care for infants with BPD after the NICU? What makes it easier/harder for families to access care? What do you see as the most vulnerable gaps in care for infants with BPD? How has your practice tried to address those gaps? How do you think we can help families understand their infant’s health?	How prepared did you feel to take care of your baby when you came home from the NICU? What might have helped you prepare better? How comfortable do you feel discussing questions about your child’s health with your primary care physician? Did you experience gaps in communication between your child’s primary care doctor and specialists? What makes it easier or harder to access specialty care like BPD clinic and eye clinic? What has helped you to feel more comfortable managing equipment and medications? How do you know who to go to, and for what? Has anything or anyone been helpful to you in coordinating your child’s care? Can you think of times you feel that you were mistreated or did not get the care you deserved?
Family impact	Do you notice issues related to family mental health, quality of life, or coping related to having infants with BPD?	How has this experience impacted your life? How has it impacted your physical and mental health? How has this experience impacted your family?

**Table 3:** Representative parent and stakeholder quotes on family-level barriers and facilitators to care

Pragmatic barriers		
Theme	Parent quotes	Stakeholder quotes
**Presence helps to secure resources**	“I just went back to work full-time about a month ago, but my job is pretty flexible where I can meet all those appointments. So to… have services come in has been fine. It’s actually much easier than if we had to pack up and go somewhere.” (Parent 13)	“You know the old saying, ‘The squeaky wheel gets the grease…’ If you’re here at the bedside more often, expressing your concerns ….those families probably… do get more touchpoints… just remembering that we need to make sure to reach out …on a regular basis …when they’re not here.” (Stakeholder 3)
**Systematic screening limitations**	“We had talked to the social worker… she asked us what do we need to get here? Do we need gas cards or … a cab to come and get us…. See, we didn’t know all of this. We could have been using this resource and …kept the car at home, and rode up here if they woulda’ signed it up like that. But they didn’t say none of that until the last minute.” (Parent 12)	“I think the other thing we always fear in screening… is what are we gonna do to help them once we have this information? Because we’re often inundated with needs… A lot of the social determinants of health are hard for us to screen for because everybody needs help here.” (Stakeholder 7)
**Complexity of needs**	“The transportation [isn’t] always reliable either… because sometimes something gets messed up on there, so we must call, reschedule your appointment or you just might get to your appointment late or something like that, and then they’re not able to see you and you have to reschedule. Driving yourself [is] not always ideal because the gas prices.. before, they were kind of outrageous.” (Parent 21)	When you’re able to assess the family, you find out that there is a lot more complexity behind it… Perhaps it’s childcare… unemployment… they are struggling financially and in a crisis, and prioritizing a medical appointment is just not possible at this time. But a lot of times… with an assessment, you’ll find out that there’s other reasons. The family has low medical literacy. They don’t understand the severity of the concern…despite learning about it. It really is dependent on each family and complex issues. (Stakeholder 2)
**Family functioning**		
**Trained caregiver support**	“It’s hard to have a social life or to go anywhere, do anything, even to go to a grocery store… You have to make sure you have somebody that knows what they’re looking at while she’s on oxygen …the little signs, if she starts to turn purple, if she’s not making noise… Just to make a simple trip to the grocery store, you have to train somebody in a crash course to take care of them.” (Parent 14)	“I find that the families who have social support… someone who’s actively involved in the baby’s care, tend to show up a lot more consistently. It doesn’t always have to be a father, but I do find that if there’s more than one caregiver coming to clinic, or they at least earmark that, “I work, and grandma takes care of the baby,” or, “Dad’s working. I’m staying home,” I find that those families tend to do better with access for us.” (Stakeholder 3)
**Isolation to reduce infectious exposures**	“My family… made sure to help out as much as they could and that included staying away from everybody so that they could only help, which was really great because we were confident… that they were maintaining social distancing, they were cleaning things and trying to keep themselves as healthy as possible so they could come and help us with the other two kids while we went to work and then went to the NICU.” (Parent 23)	“We spend a lot of time telling families hand washing, keep them healthy, but we also realize they’re going to be exposed to illnesses. It’s inevitable in childhood. I think families who have the financial means to keep their children home for the first year of life and not put them in childcare benefit from being exposed to less viral illnesses, but a lot of our families don’t have that financial ability. I struggle with that because ideally, I want them at home, but I know even for me, I wouldn’t be able to just stay at home. I would have to probably work to support my child and their needs.” (Stakeholder 5)
**Emotional toll of caregiving**	“He got discharged at, around 42 weeks so he was weeks of a newborn baby but like a super, super, super premature baby. I came home and we really didn’t have anyone in the house. We didn’t take him places. So, I think our social aspect of friends and family has taken a big hit for us. Which kind of trickles down mentally for everyone because we’re not living our lifestyles like we did a year ago, with the kids being able to have friends over; we’ve said no to a lot of social gatherings just for, you know, exposures.” (Parent 13) “Emotionally and just physically having to do the stuff that came with her conditions like changing her oxygen tank, cleaning out her oxygen converter, making sure if it’s sticky, get cleaned …took an emotional toll on me. Just by seeing and having it there and knowing she’s not a normal baby.” (Parent 11)	“I think it is definitely for some families it’s hard, and it’s stressful, ‘cause it’s a lot to handle for these families, especially if they need oxygen or other types of equipment, and it can be scary… I think there’s definitely a lot of anxiety with these babies that the families have. And they’re definitely hyper aware of certain symptoms. Or some families, I can tell they have problems sleeping through the night because they always feel like they have to go check on their baby to make sure that their baby’s breathing.” (Stakeholder 1)

**Table 4:** Representative parent and stakeholder quotes on system-level barriers and facilitators to care

Caregiver education		
Theme	Parent quotes	Stakeholder quotes
**Self-efficacy**	“It was intimidating to learn it all. It gave me anxiety. But I did it. You go into mom mode… The oxygen was very intimidating for me… Just making sure like, ‘Oh my God, did I do it right?’ … Those kind of insecurities I guess you have ‘cause you’re not familiar with doing stuff like that.” (Parent 14) “She was more ready than I was because she was here and they was telling her everything that she needed to know before she got home. And she reversed it to me. But I still, in my mind, I still was kinda shaky about doing anything to her… I feel it’s like, hey, they told you how to do it. You do it, ‘cause I feel like if I do it, I might mess up.” (Parent 12)	We throw [so much information] at families… in those couple of days of the surrounding discharge. I have had a number of families be like, “Oh yeah, I remember somebody talking to me about this, or somebody talking to me about that. But there was so much going on the day of discharge, but there was a point in the day where everybody just sounded like Charlie Brown’s teacher.” (Stakeholder 8) “[It would be easier to help families by] earlier family engagement, and actually not just family engagement but earlier parenting. And I hate to use the word allowing them to parent ‘cause that shouldn’t be a thing. But especially when you have a baby who is a very small preemie and a family feels like they’re just the bystander, somebody else is caring for their child… that that can become a barrier for some families.” (Stakeholder 8)
**Understanding health information**	“I’ve had to show my husband how to use the feeding pump, how to use the pulse ox machine. I know there’s written material, but I think a lot of people like hands-on and seeing it and touching it in real life. And looking at a manual, especially when you’re stressed because the baby’s crying, things aren’t going right, and machines alarming.” (Parent 01)	“Lots of people have challenges with health literacy and sometimes even just trusting the medical field too… I have this conversation in BPD clinic... “Oh, my baby’s fine without the oxygen. I’ve tried it at home by myself and… they don’t need it.” …Understanding and having that trust in us that we’re not just saying it because we want you to take an extra trip out this week. We really do need to see your baby for their health.” (Stakeholder 3)
**Trajectory of future needs**	“Nobody ever really took the time to say ‘she has this. This is gonna be her diagnosis, and this is what it means for the rest of her life.’ …How does this change things? What does this diagnosis mean? What do I need to watch for? …I think of chronic lung disease as being there just because she was born premature, ‘cause I don’t know what else it really means.” (Parent 23)	“After they are healthy enough to get home and they see, ‘Oh, they’re healthy enough to be off of oxygen,’ then the question always is like, ‘What’s next?’ …I have that conversation of, ‘You know what? You know, I fully expect your child to be running around on the basketball courts or soccer fields at, you know, at kindergarten, first grade. And if they’re not, if they’re the kids that sit down on the bench and it’s because they can’t breathe, don’t assume that’s because of [BPD]. You need to come back and let us know and also let the pediatricians know that too.’ We’ve had a couple families that said, ‘Oh, we just thought it was because they were born premature.’ And they’re 12 years old, and they’ve limited themselves. They’re deconditioned. They’re wheezing. We could’ve made your quality of life and your health better if we just had known. (Stakeholder 2)
**Care coordination benefits and limitations**	“When the girls have a couple appointments together, I feel like that makes it a lot easier if we can just get it done in one day, and that way we don’t have to go back-to-back to two different days… I think that’s pretty much one of the easier things about it.” (Parent 15) “‘Cause of his ROP exams, he had to go every two weeks – [that doctor] would only see patients on Tuesdays and Thursdays. And then he’d need to go see the nephrology team… and they’d only see patients on Mondays and Wednesdays. And it’s not like I could move up appointments or move them back, so it’s hard to see providers on the same day.” (Parent 1)	“Any of those barriers in getting to us, you only have to deal with one hurdle instead of three consecutive days. And these families have a lot of appointments. I don’t know how they do it. I’ll be honest with you. I’m a college-educated, well-organized person, and the thought of sometimes they’re coming three days a week to our institution, that’s a lot on anybody.” (Stakeholder 5) It’s somewhat disheartening listening to NICU families talk about all of the hurdles and hiccups and things that have happened on the outpatient side… You don’t know what you don’t know until you get home, and some families initially decline our [care coordination] program and say, “Nope, we don’t another–we don’t need another thing to do,” and then, later on, get referred to us and come back and say, “Wow, I really wish we had your program to begin with.” (Stakeholder 14)
**Role confusion between primary and specialty care**	“Her primary care doctor said he wasn’t gonna do anything or have any changes done to her until she got done with Children’s Hospital. And that’s never gonna happen because she has the shunt in her head and breathing problems, a hole in her heart, etc.” (Parent 11) “My pediatrician, when she called down that day… she’s wondering what she should do to help us, and they’re like ‘well, she’s not a patient of ours ‘cause we haven’t seen them yet….’ Where do we go? What do we do? It was my pediatrician on the phone with them trying to figure out what to do.” (Parent 24)	“Some parents are like, ‘Well, they won’t make it to pulmonology to figure out their oxygen situation. Can this baby be weaned off of oxygen?’ They need pulse oximetry for that to be done, right? And that’s something that I can’t do as a pediatrician. I’ve had parents just taking off. They’ll just be like, ‘Oh, well, let’s see how they do at home.’ And they’ll just wean off their kid’s own oxygen or no longer have an oxygen order. And so the kid is just done with their oxygen, right? …What do you do as a pediatrician?” (Stakeholder 11)
**Opportunities and limitations of support services**	“I knew that from the home care agency we had a nurse coming to the house, and so I felt supported... I’ll be honest. At first, I didn’t think I was gonna need the home care nurse, but I was so grateful to have her, because she would help me pick up on things, and not maybe feel so overwhelmed.” (Parent 1) “In-home therapy has helped tremendously because when she was starting she doesn’t even know she has legs, seriously, and now she’s walking and they recommended the prosthesis for her feet and everything.” (Parent 5)	“How-to videos [are] on our website… [in a] third to fifth grade [reading level] so that the voice-over for that how-to video would be accessible to a family member. So if they’re having a problem with the suction machine and they wanna troubleshoot it or how do you apply an oximeter probe, that they could go and have that reinforcement to them 24/7. And it’s there free on our website to anybody in the world, whether they’re our patient or not. That is something that we’ve been able to provide just based on a commitment to safe care in the home.” (Stakeholder 18) “Some families do not engage in all this and there are systemic barriers… where families fear that if you have someone coming into your home on a week-to-week or month-to-month basis, even if it is designed as a supportive presence, there is a deep-rooted fear for some of these families that it will be used against them in the future, that if they acknowledge a need, that it could be used against them, whether it’s with Child Protection Services or other consequences.” (Stakeholder 2)

## Abbreviations

BPD: bronchopulmonary dysplasia
NICU: neonatal intensive care unit
